# Supplementary material for: Causal Relationship between Aging and Anorexia Nervosa: A White-Matter-Microstructure-Mediated Mendelian Randomization Analysis
Source: Biomedicines. 2024 Aug 16;12(8):1874. doi: 10.3390/biomedicines12081874 (PMC11351342; doi:10.3390/biomedicines12081874)
Supplement: Supplementary file 1 [file biomedicines-12-01874-s001.zip › Supplementary Figure/Figure S4/Singe SNP plot of MR analysis for TL to ubm-b-1544.pdf]

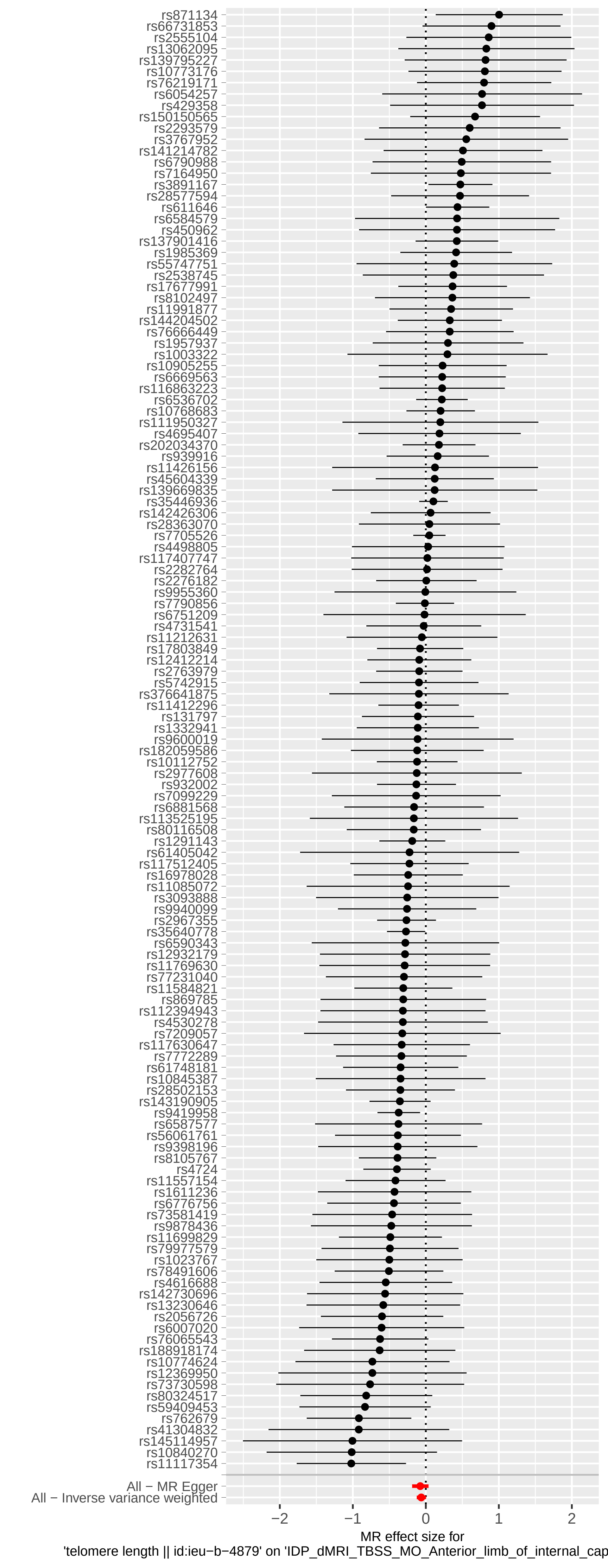
rs871134
rs66731853
rs2555104
rs13062095
rs139795227
rs10773176
rs76219171
rs6054257
rs429358
rs150150565
rs2293579
rs3767952
rs141214782
rs6790988
rs7164950
rs3891167
rs28577594
rs611646
rs6584579
rs450962
rs137901416
rs1985369
rs55747751
rs2538745
rs17677991
rs8102497
rs11991877
rs144204502
rs76666449
rs1957937
rs1003322
rs10905255
rs6669563
rs116863223
rs6536702
rs10768683
rs111950327
rs4695407
rs202034370
rs939916
rs11426156
rs45604339
rs139669835
rs35446936
rs142426306
rs28363070
rs7705526
rs4498805
rs117407747
rs2282764
rs2276182
rs9955360
rs7790856
rs6751209
rs4731541
rs11212631
rs17803849
rs12412214
rs2763979
rs5742915
rs376641875
rs11412296
rs131797
rs1332941
rs9600019
rs182059586
rs10112752
rs2977608
rs932002
rs7099229
rs6881568
rs113525195
rs80116508
rs1291143
rs61405042
rs117512405
rs16978028
rs11085072
rs3093888
rs9940099
rs2967355
rs35640778
rs6590343
rs12932179
rs11769630
rs77231040
rs11584821
rs869785
rs112394943
rs4530278
rs7209057
rs117630647
rs7772289
rs61748181
rs10845387
rs28502153
rs143190905
rs9419958
rs6587577
rs56061761
rs9398196
rs8105767
rs4724
rs11557154
rs1611236
rs6776756
rs73581419
rs9878436
rs11699829
rs79977579
rs1023767
rs78491606
rs4616688
rs142730696
rs13230646
rs2056726
rs6007020
rs76065543
rs188918174
rs10774624
rs12369950
rs73730598
rs80324517
rs59409453
rs762679
rs41304832
rs145114957
rs10840270
rs11117354
All − MR Egger
All − Inverse variance weighted
−2
−1
0
1
2
MR effect size for
'telomere length || id:ieu−b−4879' on 'IDP_dMRI_TBSS_MO_Anterior_limb_of_internal_cap
